# Efficiency Analysis of Disruptive Color in Military Camouflage Patterns Based on Eye Movement Data

**DOI:** 10.3390/jemr18040026

**Published:** 2025-07-02

**Authors:** Xin Yang, Su Yan, Bentian Hao, Weidong Xu, Haibao Yu

**Affiliations:** 1 Key Laboratory of National Defense Science and Technology, Army Engineering University, Nanjing 210007, China; yangxin@aeu.edu.cn (X.Y.); wikings@aliyun.com (S.Y.); xweibing1968@aliyun.com (W.X.); 13369829295@163.com (H.Y.); 2 Field Engineering College, Army Engineering University, Nanjing 210007, China

**Keywords:** disruptive color, camouflage design, eye movement analysis, camouflage effect, camouflage pattern, target concealment

## Abstract

Disruptive color on animals’ bodies can reduce the risk of being caught. This study explores the camouflaging effect of disruptive color when applied to military targets. Disruptive and non-disruptive color patterns were placed on the target surface to form simulation materials. Then, the simulation target was set in woodland-, grassland-, and desert-type background images. The detectability of the target in the background was obtained by collecting eye movement indicators after the observer observed the background targets. The influence of background type (local and global), camouflage pattern type, and target viewing angle on the disruptive-color camouflage pattern was investigated. This study aims to design eye movement observation experiments to statistically analyze the indicators of first discovery time, discovery frequency, and first-scan amplitude in the target area. The experimental results show that the first discovery time of mixed disruptive-color targets in a forest background was significantly higher than that of non-mixed disruptive-color targets (t = 2.54, *p* = 0.039), and the click frequency was reduced by 15% (*p* < 0.05), indicating that mixed disruptive color has better camouflage effectiveness in complex backgrounds. In addition, the camouflage effect of mixed disruptive colors on large-scale targets (viewing angle ≥ 30°) is significantly improved (F = 10.113, *p* = 0.01), providing theoretical support for close-range reconnaissance camouflage design.

## 1. Introduction

Camouflage technology can control the salient features of the target surface and reduce the detectability of a target; it is widely used in the field of military target camouflage protection by countries all over the world. The traditional view is that the spots in the camouflage patterns should be consistent with the spots in the background [1]. Spots refer to areas of color blocks of different colors, shapes, or sizes. Most of the research on camouflage patterns on moving targets is based on this basic principle. Some scholars call a similar principle ‘background matching’ [2]. In addition to this, disruptive color is a different camouflage principle. Caro et al. [3] first noticed that the camouflage principle of disruptive color and background matching is different. Many subsequent scholars [4,5] have performed research on the mechanisms and application methods of disruptive color. In 2009, Stevens and Merilaita reviewed previous studies, and defined disruptive color as a group of spots that can produce false edges and boundaries, hindering the detection and recognition of the true con tour and shape of the whole target or part of the target [6].

Currently, the understanding and research on disruptive color’s effect mainly come from animal body colors [7]. Hanlon et al. [8] tested thousands of images of camouflaged cephalopods (squid, etc.) in the wild. They showed that background size, contrast, and edges are the keys to the formation of camouflage patterns, and background matching is often used in conjunction with disruptive color. Kang et al. [9] used a bird vision model to study how background matching and disruptive color mechanisms are used to select a perching tree background and the body direction of two species of moths. Quantitative research by Price [10] showed that the body color of seaside crabs plays a dominant role in background matching and disruptive color under different environmental backgrounds. Sharman et al. [11] separated the influence of disruptive color on target positioning and recognition. She found that disruptive color not only hindered target detection, but also increased recognition time. In addition to exploring the static effect of disruptive color, Stevens et al. [12] showed that targets with high-contrast stripes were found less frequently in motion than targets with background-matching spots when moving. In contrast, the opposite conclusion was found when stationary.

**Figure 1:**
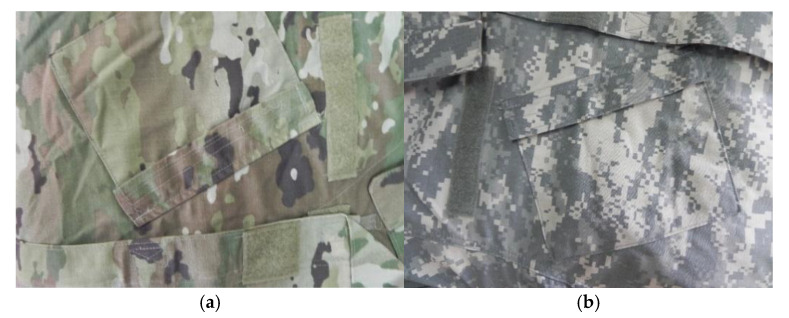
Examples of the camouflage patterns of a multi-terrain camouflage uniform. (**a**) The British army’s multi-terrain camouflage. (**b**) The US military’s MARPAT digital camouflage.

Due to the necessity for military secrets, there is little research on the applied theory of disruptive color in the military field. However, based on the camouflage pattern effect of some equipment designs, the concept of disruptive color is already applied to a certain extent. In the Dual-Tex camouflage uniform developed in 1977, the U.S. military used rare colors, such as blue and brown, in the background. The newly developed MARPAT (marines developed their own digitalized marine pattern) digital camouflage has four primary colors: brown, black, and other primary colors form a high internal contrast and produce the disruptive color effect [13]. CADPAT (a digitalized pattern called Canadian pattern) digital camouflage, designed by Canada [14], achieved the best results in tests conducted by various NATO (North Atlantic Treaty Organization) troops. Its woodland camouflage even uses up to 31% black color to form a high contrast. The British army’s multi-terrain camouflage improves its applicability to different backgrounds by superimposing the disruptive color with a high degree of layering and contrast on the base color. A certain proportion of brown and black was also used in the small-spotted camouflage uniforms used by the German army [15]. Figure 1 shows the multi-terrain camouflage uniform of the British military, which applies the principle of disruptive color to a certain extent.

Current research further reveals a correlation between camouflage effects and eye movement behavior. For example, Lin et al. [16] demonstrated, using eye tracking technology, that the first fixation time and scanning amplitude of a target can effectively quantify its visual salience, providing a new method for evaluating camouflage effectiveness. However, these studies mainly focus on animal camouflage or static artificial backgrounds, and the special needs of military targets have not been systematically explored.

In addition, most theoretical studies are based on artificial mottled backgrounds and animal visual systems, and whether the conclusions can be applied to natural backgrounds deserves further study. This paper applies the disruptive color method to the simulated camouflage target. Based on the eye movement observation index [17,18,19,20,21], the efficiency improvement of disruptive color under different backgrounds and observation scales is discussed. It lays a foundation for the application of disruptive color in military camouflage.

The background-matching principle requires that the color [22], brightness [23], texture [24,25], and other characteristics of camouflage should be consistent to reduce the salience of the target in the background [26,27,28]. From a psychophysical perspective, disruptive coloration operates by impeding Gestalt contour integration. Eye movement indicators, such as first saccade amplitude, directly reflect the failure of peripheral vision to group fragmented edges, while first fixation time correlates with cognitive load during the feature-binding stages. On the one hand, it hides the real edge, and on the other hand, it generates false contours in the target to attract the observer’s attention. At present, there is no clear concept to distinguish whether a pattern contains a disruptive color effect or not. Stevens pointed out that the following five sub-principles should be considered when selecting the disruptive color: (1) differential color mixing: spots with different color characteristics work together [29], some of which are consistent with the spots in the background; (2) maximize the disruptive contrast, which should form a strong contrast between adjacent spots, destroying the shape of the target [30,31]; (3) the disruptive-color spots should be as close as possible to the contour of the target; (4) surface damage, i.e., creating a significant false edge inside the target; and (5) continuous damage, i.e., covering continuous patterns on the discontinuous parts of the target, reducing the exposure characteristics of significant parts.

Considering that the application of eye movement analysis in the field of military camouflage is still in the exploratory stage, the existing mixed-color theory is mostly based on small-scale biological backgrounds, and whether its conclusions are applicable to large-scale military targets is still unclear. The synergistic effect of mixed colors with digital camouflage and large-spot camouflage has not been empirically tested. This study considers that deformation camouflage needs to be applicable to multiple backgrounds, and uses black spots as mixed colors for testing experiments. Firstly, selecting the main color that matches the background usually has a high brightness coefficient, and black can form a high brightness contrast with other main colors; it can form a high brightness contrast with the main background colors, such as forest green and desert green, where Δ L > 30. Secondly, when looking down at the background image, it usually contains certain black shadow spots, which can simulate natural shadow features and also conform to military camouflage’s design conventions. Therefore, the setting of black conforms to the first sub-principle of disruptive color setting. In addition, when designing camouflage training uniforms, a certain proportion of black is usually used in the design of military camouflage uniforms in various countries. By designing multi-background and multi-scale eye tracking experiments, the dynamic effectiveness of mixed disruptive colors in military camouflage is here systematically evaluated for the first time, filling the theoretical and application gaps mentioned above.

## 2. Methods

### 2.1. Experimental Materials and Environment

The aerial images of typical woodland-type, desert-type, and grassland-type backgrounds were collected, and the ground resolution of the background image was controlled to be 10 cm. According to the literature [32,33], large-spot camouflage patterns and digital camouflage patterns were designed, respectively, in which the number of primary colors of camouflage pattern was 3. Select the combination of the transport vehicle template and the camouflage pattern design as the target. According to the above principles of disruptive-color design, black spots were placed into the target image to form a contrasting target with disruptive color. The results are shown in Table 1. The background image was obtained using vertical angle optical imaging of the UAV (Unmanned Aerial Vehicle). The background image data of woodland and grassland types are from Southeast China and Southeast China, and the background image data of desert type are from Northwest China. The image’s ground sampling distance (GSD) shall be 10 cm. The target image was randomly placed in the background to form the observation material. The viewing angle of the target was determined by the observation distance and the set size of the target.

The tobii T120 eye tracker was used to record the data in the experiment, and the sampling frequency was 60 Hz. According to the screen size, set the viewing distance so that the viewing angle is within 60°. The screen resolution is 1920 × 1080. A total of 32 people participated in the experiment. The participants voluntarily registered through a campus announcement, all of whom were enrolled students aged 22–30 with corrected vision of 5.0 or above. All participants had no disguised professional background to ensure the universality of the experimental results. All participants were free of visual impairment or were corrected for visual impairment. Each participant in the experiment was provided with an explanation of the experimental process to ensure that they maintained their optimal state in each experiment. After each participant completed a single experiment, they were required to take a 10 min break. The laboratory environment had constant light and no noise interference. When there was fatigue or distraction, and attention was not focused, we paused the experiment until the participants completely relaxed, before continuing. In the experiment, the participants’ heads were fixed using the chin rest embedded in the Tobii T120, ensuring that the accuracy of eye movement data collection was less than 0.5° of the viewing angle.

### 2.2. Experimental Contents

**Figure 2:**
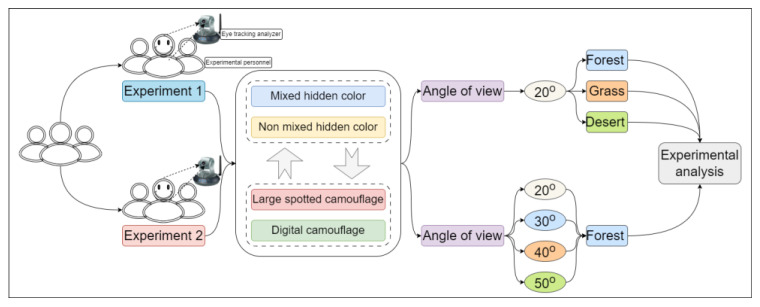
Overall flow chart of the experiment.

**Figure 3:**
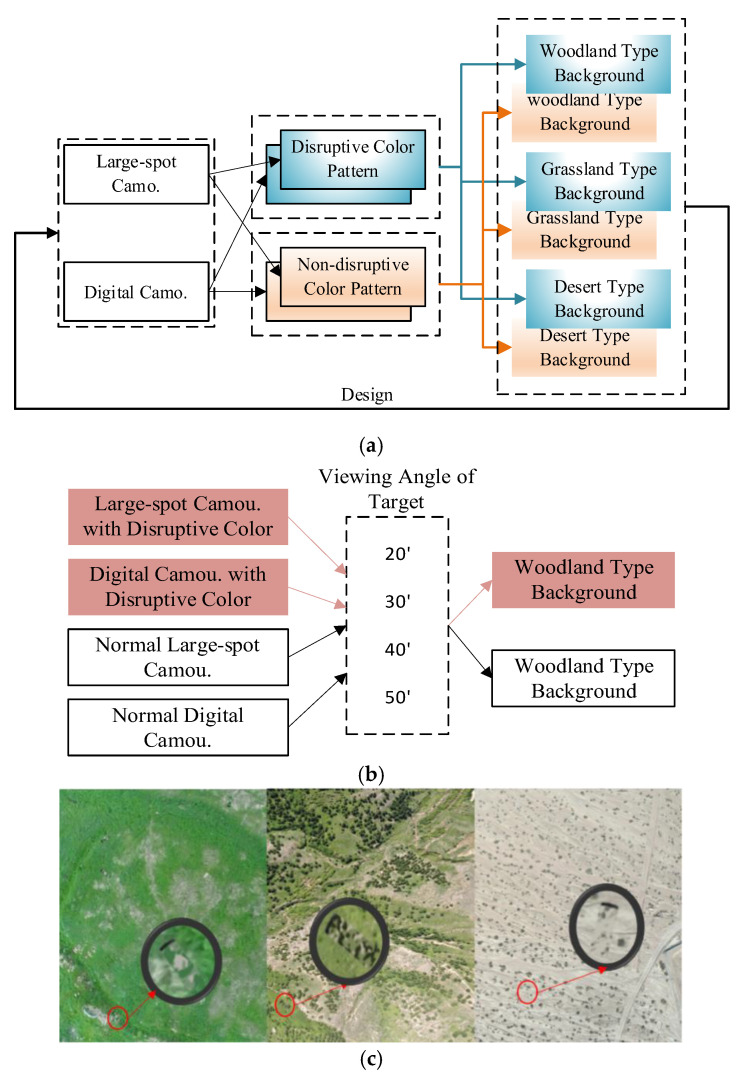
Composition of a group of observation experiment materials. (**a**) Schematic of material generation method in Experiment 1. (**b**) Schematic of the material generation method of experiment 2. (**c**) Three backgrounds (partial backgrounds) and target placement examples used in the observation experiment.

The overall structure diagram of the experiment is shown in Figure 2, where two sets of experiments were set up separately. In Experiment 1, the perspective of all targets was set to $20^{\prime }$, and three backgrounds, including forest, grassland, and desert, digital camouflage, large-spot camouflage, and mixed-color effects, were tested at different positions. Experiment 2 only changed the perspective while keeping the other conditions constant. Two experiments were set up, respectively. In experiment 1, the viewing angles of all targets were set as $20^{\prime }$. The effects of different backgrounds, different camouflage designs, and different positions of disruptive color were tested. The setting method and experimental process of each type of background experiment are entirely consistent. We randomly placed the corresponding 4 digital camouflage targets and 4 large-spot camouflage targets into the corresponding background, and placed the target pattern with disruptive color at the same position and angled in another image with the same background as a control experiment (as shown in Figure 3a). Experiment 2 was conducted in a woodland-type setting. The target size was set to meet the viewing angles of $20^{\prime }$, $30^{\prime }$, $40^{\prime }$, and $50^{\prime }$, respectively, and the target was covered with either large-spot or digital camouflage. The target with non-disruptive color was randomly placed in the woodland background, and another target pattern with disruptive color was set in the same position and angle of the same background to form a control experiment to test the effect of disruptive color at different viewing angles (as shown in Figure 3b). In experiment 1, 6 observation images were taken, including 3 types of background. In experiment 2, there were two observation images. No more than 8 targets were randomly set in each image, and a black screen was inserted to form a visual buffer when switching between images. In the experiment, the participants’ heads were fixed using the chin rest embedded in the Tobii T120, ensuring that the accuracy of eye movement data collection was less than 0.5° of viewing angle. The materials composed of the three backgrounds and targets of the experiment are shown in Figure 3c.

**Figure 4:**
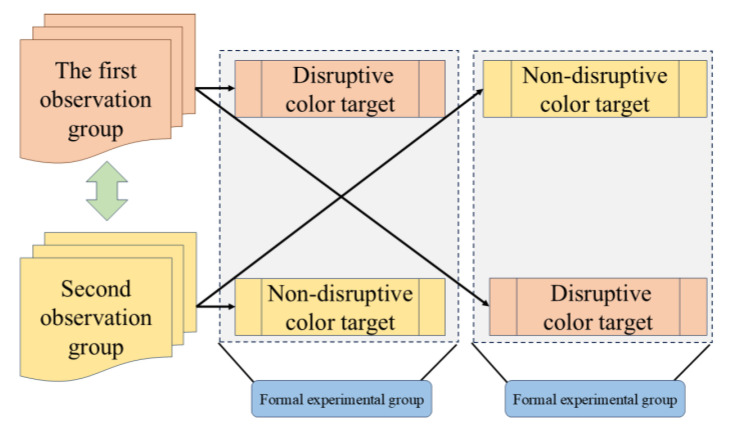
Observation task flow of two groups of participants.

Setting up an utterly similar control group can eliminate the effect of other distractions. However, subjects observing the control experiment at the same time will introduce prior information for eye-tracking experiments, which will induce a large error. The first group observed non-disruptive-color targets, and the second group observed disruptive-color targets. The first group of participants observed the disruptive-color targets in the formal experimental group and the non-disruptive-color targets in the backup experimental group. The second group of participants observed the non-disruptive-color targets in the formal experimental group and the disruptive-color targets in the backup experimental group. The experimental process is shown in Figure 4.

Before the experiment, the participants needed to understand the target to be searched and the various camouflage patterns attached in advance. Then, after adjusting their sitting posture, the eye movement error was calibrated. The materials from experiment 1 and experiment 2 were randomly disordered. Each observer observed a background image with a target of 50s and left-clicked on the detected target with the mouse. Before each experimental image was presented, a pure black pattern was displayed for 5s to form a visual buffer. In the experiment, the eye tracker simultaneously recorded the eye movement coordinate data and the position of the left click of the mouse. Each experimenter was required to observe one set of disruptive-color targets and another set of non-disruptive-color targets.

### 2.3. Analytical Method

The eye tracker mainly records the coordinates of the gaze position of the observer’s eye when searching for a target. After the secondary processing of the data, indexes, such as the time of first discovery of a single target, the discovery frequency, and the first saccade amplitude of the target area, were obtained. The first discovery time of the target refers to the time spent by the participants from the beginning of observing the background material to the first determination of the target’s position. It should be noted that, although some saccades data are on the target, these saccades are random, and the participants did not find the target. Therefore, this index data needs to be calibrated with the click-hit time of the target. Target discovery frequency is the number of target clicks divided by the total number of observations. These two indexes are related to the saliency of the target. The lower the discovery frequency, the greater the difficulty of discovery. The shorter the first discovery time, the lower the difficulty of target detection. The first saccade amplitude of the target refers to the saccade distance before the target is detected. Studies have shown [34,35] that the larger the index value, the more pronounced the target.

Data and results analysis can be performed in two statistical ways. The paired *t*-test was applied to targets in disruptive-color and non-disruptive-color control experiments with the same location and angle. An independent *t*-test was applied to targets with different camouflages. A significance lower than 0.05 was considered a significant difference. One-way ANOVA (Analysis of Variance) was applied to the effect analysis of the scale factors in experiment 2, and LSD (Least Significant Difference) postmortem analysis was used to distinguish the significance of differences within groups.

## 3. Results

**Figure 5:**
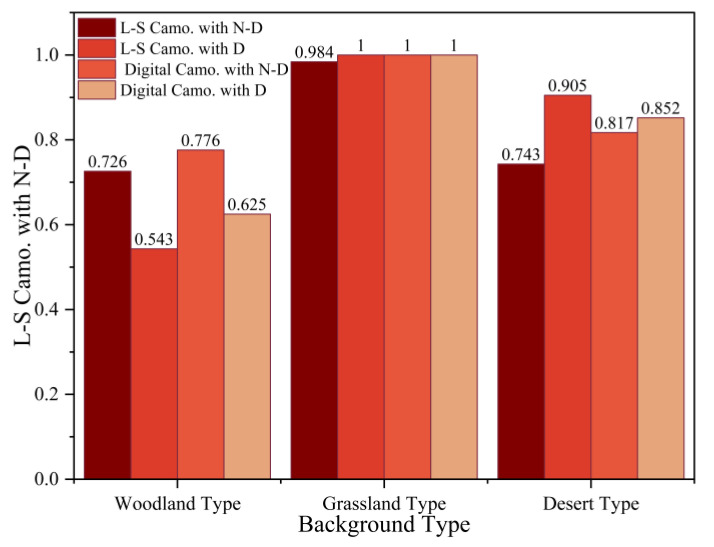
The proportion of correctly clicking on four types of targets in different backgrounds.

In experiment 1, the click frequency of the target was analyzed first. Targets of the same type were treated as a set of samples, and the histogram results were produced and shown in Figure 5. The abscissa is three different types of backgrounds. The four different colored bars represent the large-spot camouflage with non-disruptive color targets (L-S Camo. with N-D.), the large-spot camouflage with disruptive color targets (L-S Camo. with D.), the digital camouflage with non-disruptive color targets (Digital Camo. with N-D.), and the digital camouflage with disruptive color targets (Digital Camo. with D.). The ordinate is the click frequency of the target. The results show that woodland backgrounds were clicked less frequently than the other two types of backgrounds. Targets are almost 100% detectable in grassland-type backgrounds. In addition, the click rate of the non-disruptive color targets in woodland-type backgrounds was higher than that of the disruptive-color targets. In comparison, the click rate of the non-disruptive-color targets in the grassland-type backgrounds is slightly lower than that of the disruptive-color targets. There was no significant difference in click rate between large-spot camouflage and digital camouflage.

The first discovery time and the first saccade amplitude indexes of a single target were analyzed. The index mean value of a single target was regarded as a sample, and there were a total of eight disruptive-color and non-disruptive-color control targets at different positions in different backgrounds, and the paired *t*-test results were calculated. In the grassland-type background, there was no significant difference between the mean time of first discovery (t = −0.386, *p* = 0.711 > 0.05) and the mean first saccade amplitude indexes (t = −0.77, *p* = 0.465 > 0.05). In the desert-type background, the mean first discovery time (t = 2.77, *p* = 0.028 < 0.05) was significantly different, and the mean value of non-disruptive color was 3.39 higher than that of the disruptive color. The mean first saccade amplitude index (t = −1.381, *p* = 0.210 > 0.05) was not significantly different. In the woodland-type background, the two indexes were significantly different. Among them, the mean value of the mean first discovery time index (t = −2.54, *p* = 0.039 < 0.05) of disruptive color was 3.84s higher than that of the non-disruptive color. The mean value of the mean first saccade amplitude index (t = 2.455, *p* = 0.044 < 0.05) of disruptive color is 2.90s lower than that of the non-disruptive color. The 3.84s prolongation of first fixation time (t = 2.54, *p* = 0.039) under woodland backgrounds reflects increased cognitive load in contour segmentation. Disruptive spots disrupted global shape processing, forcing serial search rather than parallel pop-out (Figure 5).

In the above paired *t*-test, the correlation *p* of paired samples was greater than 0.05, suggesting differences among individuals. Three indexes of a single control target in a woodland-type background were investigated. The results show that, when the target close to the complex woodland background was added with disruptive color, the index display was not conducive to observation. After the target near the monotonous loess background was added with disruptive color, the index display can improve the observation efficiency.

Taking all large-spot camouflages and digital camouflages under the same background as two groups of samples, an independent *t*-test of the three indexes was analyzed, and the results are shown in Table 2. The $t^{\prime }$ and $p^{\prime }$ in the table indicate that the Levene variance of the sample data was tested for heterogeneity and corrected. In the grassland-type backgrounds, the click frequency of most targets is 1.0, resulting in the non-homogeneity of variance. In addition, the three indexes of the grassland-type background and woodland-type background all showed no differences between large-spot camouflage and digital camouflage. There were differences in the mean time of the first discovery index in the desert-type background, but the *p*-value was very close to 0.05, and the *p*-value of the mean index of the first saccade amplitude was slightly higher than 0.05. The click frequency index *p*-value was higher than 0.05, and this shows that two of the three indexes indicate that there is a slight difference between the digital camouflage and the large-spot camouflage in the desert-type background, and the other index indicates that there is no difference.

Experiment 2 investigated the effects of different viewing angles of disruptive-color targets. First, using viewing angles as an influencing factor, a one-way ANOVA was used to study whether the influence of viewing angles on the three indexes was significant. The results show that the impact on click frequency (F(3,12) = 6.626, *p* = 0.007 < 0.05) is significant. LSD postmortem analysis shows significant differences between target viewing angle of $20^{\prime }$ and $30^{\prime }$, $20^{\prime }$ and $40^{\prime }$, and $20^{\prime }$ and $50^{\prime }$. The differences in the indexes from other viewing angles are not significant. Their influence on the mean time of the first discovery (F(3,12) = 10.813, *p* = 0.01 < 0.05) is significant. LSD postmortem analysis shows significant differences between the target viewing angles of $20^{\prime }$ and $40^{\prime }$, $20^{\prime }$ and $50^{\prime }$, and $30^{\prime }$ and $50^{\prime }$. The differences in the indexes from other viewing angles are not significant. Their influence on the mean value of the first saccade amplitude (F(3,12) = 10.579, *p* = 0.01 < 0.05) is significant. LSD postmortem analysis shows significant differences between the target viewing angles of $20^{\prime }$ and $40^{\prime }$, $20^{\prime }$ and $50^{\prime }$, and $30^{\prime }$ and $50^{\prime }$. The differences in the indexes from other viewing angles are not significant.

**Figure 6:**
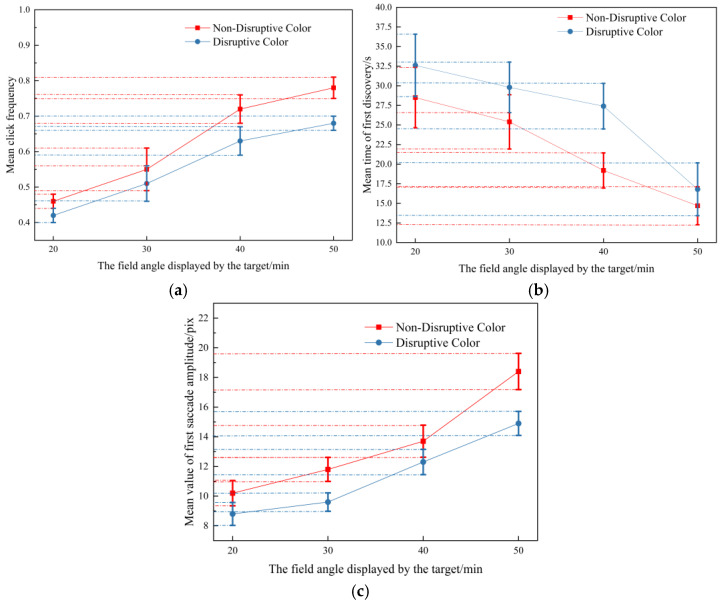
Mean value curve of three indexes of four types of targets with different field angles. (**a**) Mean value of target click frequency at different scales. (**b**) Mean time of first discovery of targets with different scales. (**c**) Mean value of first saccade amplitude of targets with different scales.

The mean values of the three indexes of disruptive color and non-disruptive color are plotted as curves, respectively. The results are shown in Figure 6. The upper and lower bars in the figure represent this data group’s standard deviation. The uniform rule of three indexes for the mean value of all the disruptive-color and non-disruptive-color targets shows that the observation difficulty of disruptive-color targets is higher than that of non-disruptive-color targets. Moreover, the bigger the target, the easier it is to find. According to the overlap of some standard deviation bars, individual targets in the sample have different performances, which indicates that the effect produced by the disruptive color is most likely related to the location and background of the target. In addition, by observing the trend in the mean value of the target click frequency from all viewing angles, the mean value of the first discovery time of the target of $20^{\prime }$, $30^{\prime },$ and $40^{\prime }$, and the mean value of the first saccade amplitude of the target of $20^{\prime }$, $30^{\prime }$ and $50^{\prime }$, it can be found that the disruptive-color and non-disruptive-color contrast will make the index value of the target with a larger viewing angle change more.

## 4. Discussion

In experiment 1, data analysis of the three indexes shows the different effects of disruptive- and non-disruptive-color targets in three backgrounds. In woodland-type backgrounds, the camouflage effect of disruptive-color targets is better than that of non-disruptive-color targets. In desert-type backgrounds, the disruptive-color target is slightly worse than the non-disruptive-color target. In grassland-type backgrounds, there is no significant difference between the camouflage effect of the disruptive-color and non-disruptive-color targets, and both targets can be quickly captured by the participants. Our analysis is that the grassland type includes almost monotonous backgrounds, and the uniform color area in the background is large. Both types of targets can be found quickly, and the click frequency index is almost 1.

This study found that mixed disruptive colors significantly improved camouflage effectiveness in forest backgrounds (with a 15% increase in first discovery time, *p* < 0.05), while reducing effectiveness in desert backgrounds (with a 3.39 s decrease in first discovery time, *p* = 0.028). The 15% reduction in click frequency and 2.90° decrease in first saccade amplitude collectively demonstrate two psychophysical effects. Edge disruption effect: High-contrast spots induced false contour grouping (evidenced by reduced saccade amplitude), delaying peripheral detection Cognitive masking effect: Prolonged fixation time indicates serial attentional scanning when global shape cues are invalidated (FIT Stage 2 processing). This dual mechanism explains why disruptive coloration outperforms background matching only in high-complexity scenes where Gestalt principles dominate. This result is highly consistent with the conclusion of Sharman et al.: when the internal contrast of the target is higher than the background (such as black spots in the desert and light sand), the camouflage effect will be weakened. However, unlike the “edge destruction” mechanism proposed by Stevens this study further reveals that the effectiveness of mixed colors depends not only on local edge interference, but also on the complexity of the global background. The natural high-contrast areas formed by tree shadows in forest land (Δ L > 40) provided a “visual fusion” environment for mixed-color spots, while the homogeneous background in desert (Δ L < 15) amplifies the abruptness of mixed colors. This provided a new principle for military camouflage design: the application of mixed disruptive colors requires a priority evaluation of the global contrast distribution of the background.

Unlike traditional subjective scoring methods, this study reveals the dual mechanism of mixed colors through a joint analysis of first-glance amplitude (reflecting target significance) and first discovery time (reflecting search efficiency): it not only reduces the significance of the target (reducing glance amplitude by 2.90, *p* = 0.044), but also prolongs the cognitive load of the search process (increasing discovery time by 3.84 s, *p* = 0.039). This method complements the currently proposed “dynamic gaze entropy” model, and establishes a new paradigm for objectively quantifying military camouflage effectiveness.

Therefore, the indexes of the two targets show no differences. In the desert-type background, the contrast of disruptive color inside the target is higher than that of spot brightness in the background, so the disruptive-color target is more obvious in the background. This is consistent with the research of Lin et al. [36]—that is, the contrast in the target higher than the background is not conducive to camouflage. In the woodland-type background, the disruptive-color targets produced better results. This is mainly due to the low-brightness shadow spots caused by the height differences of trees in the woodland-type background, resulting in high brightness contrast in the background, which increases the complexity of the background. It can be seen that whether or not disruptive color can produce a better camouflage effect than pure background matching is related to background. In fact, for natural backgrounds, background complexity is closely related to observation resolution. Some of the literature [37,38] studied the background scale corresponding to small-sized animals, such as moths, which is different from the background scale and background complexity corresponding to military targets. Therefore, the characteristics of the overall background should be carefully studied when determining the disruptive color.

On the other hand, both experiments show that the target detectability at different positions in the same background (woodland-type backgrounds) varies. That is, the camouflage performance of the target is highly correlated with its surrounding background. Therefore, selecting a regional background for camouflage plays a very important role. The disruptive color plays a greater role in more complex backgrounds. Furthermore, the brightness of the background is entirely derived from solar radiation. Except for artificial targets, most of the high-brightness contrast in the background comes from shadows. This indicates that the influence of shadowed areas should be considered when studying the background primary color of the whole area.

Experiment 2 shows that the disruptive color improves the camouflage performance of large-scale targets. Compared with background matching, the disruptive color can produce a better camouflaging effect when observed closer. Therefore, when testing the efficiency of disruptive-color spots, it should be ensured that the target has a larger viewing angle. In addition, there is almost no significant difference between the three indexes of digital camouflage and large-spot camouflage. Theoretically, the effectiveness enhancement of digital camouflage compared to large-spot camouflage is limited, and the viewing angle of $20^{\prime }$ is so small that it is almost impossible to distinguish subtle differences. At the same time, it is shown that the choice of background location has a more significant effect on the camouflaging effect than the spot type.

## 5. Conclusions

In this paper, the effect of disruptive color on camouflage is studied using an eye movement analysis. Two groups of disruptive-color and non-disruptive-color control experiments were designed. The first group of control experiments included three types of backgrounds and two randomly placed camouflage simulation targets of the same size. The second group of control experiments included camouflage simulation targets of different viewing angles on the same woodland background. The first discovery time, discovery frequency, and the first saccade amplitude of the target area were counted using eye movement observation experiments. The results show that disruptive color was better than non-disruptive color on the woodland type background, whereas the opposite effect was found in the desert type background, and there was no statistical difference between the two on the grassland type background. The results indicate whether disruptive color can induce a better camouflage effect is related to the complexity of local and global backgrounds. On the other hand, the effect of disruptive-color camouflage on the eye movement index value of large-scale targets is more significant. This shows that disruptive-color camouflage can improve the camouflaging effect of close-range reconnaissance. The research in this paper lays a foundation for the application of disruptive color in camouflage.

## Figures and Tables

**Table 1:** Simulated camouflage target pattern under three backgrounds.

		Woodland Type	Grassland Type	Desert Type
Non-disruptive color camouflage	Digital camouflage	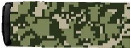	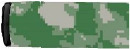	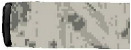
	Large-spot camouflage	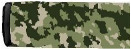	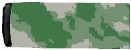	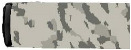
Disruptive color camouflage	Digital camouflage	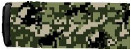	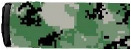	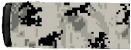
	Large-spot camouflage	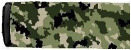	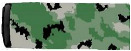	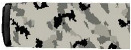

**Table 2:** Independent *t*-test results of three eye movement indexes of large-spot camouflage and digital camouflage.

	Click Rate	Mean Value of the First Discovery Time	Mean Value of the First Saccade Amplitude
Grassland type	$t^{\prime }=1.426$ $p^{\prime }=0.197>0.05$	t=1.572 p=0.138>0.05	t=0.345 p=0.735>0.05
Desert type	t=−0.739 p=0.473>0.05	t=2.187 p=0.046<0.05	t=2.008 p=0.064>0.05
Woodland type	t=1.831 p=0.088>0.05	t=−1.392 p=0.186>0.05	t=1.056 p=0.309>0.05

Note: The $t^{\prime }\;$ and $p^{\prime }$ indicate that the Levene variance of the sample data is tested for heterogeneity and corrected.

## Data Availability

The data are not publicly available due to privacy or ethical restrictions.
